# The Risk of Stroke after Percutaneous Vertebroplasty for Osteoporosis: A Population-Based Cohort Study

**DOI:** 10.1371/journal.pone.0031405

**Published:** 2012-01-31

**Authors:** Ching-Lan Wu, Jau-Ching Wu, Wen-Cheng Huang, Hung-Ta H. Wu, Hong-Jen Chiou, Laura Liu, Yu-Chun Chen, Tzeng-Ji Chen, Henrich Cheng, Cheng-Yen Chang

**Affiliations:** 1 Department of Radiology, Taipei Veterans General Hospital, Taipei, Taiwan; 2 Department of Neurosurgery, Neurological Institute, Taipei Veterans General Hospital, Taipei, Taiwan; 3 School of Medicine, National Yang-Ming University, Taipei, Taiwan; 4 Institute of Pharmacology, National Yang-Ming University, Taipei, Taiwan; 5 Department of Ophthalmology, Chang Gung Memorial Hospital, Taoyuan, Taiwan; 6 College of Medicine, Chang Gung University, Taoyuan, Taiwan; 7 Department of Medical Research and Education, National Yang-Ming University Hospital, I-Lan, Taiwan; 8 Institute of Hospital and Health Care Administration, National Yang-Ming University School of Medicine, Taipei, Taiwan; University of Modena and Reggio Emilia, Italy

## Abstract

**Purpose:**

To investigate the incidence and risk of stroke after percutaneous vertebroplasty in patients with osteoporosis.

**Methods:**

A group of 334 patients with osteoporosis, and who underwent percutaneous vertebroplasty during the study period, was compared to 1,655 age-, sex- and propensity score-matched patients who did not undergo vertebroplasty. All demographic covariates and co-morbidities were deliberately matched between the two groups to avoid selection bias. Every subject was followed-up for up to five years for stroke. Adjustments using a Cox regression model and Kaplan-Meier analyses were conducted.

**Results:**

A total of 1,989 osteoporotic patients were followed up for 3,760.13 person-years. Overall, the incidence rates of any stroke, hemorrhagic stroke and ischemic stroke were 22.6, 4.2 and 19.6 per 1,000 person-years, respectively. Patients who underwent vertebroplasty were not more likely to have any stroke (crude hazard ratio = 1.13, *p* = 0.693), hemorrhagic stroke (HR = 2.21, *p* = 0.170), or ischemic stroke (HR = 0.96, *p* = 0.90). After adjusting for demographics, co-morbidities and medications, the vertebroplasty group had no significant difference with the comparison group in terms of any, hemorrhagic and ischemic strokes (adjusted HR = 1.22, 3.17, and 0.96, *p* = 0.518, 0.055, and 0.91, respectively).

**Conclusions:**

Osteoporotic patients who undergo percutaneous vertebroplasty are not at higher risk of any stroke in the next five years after the procedure.

## Introduction

Stroke is a major cause of disability and death worldwide [1], [2]. Osteoporosis, as a known consequence of stroke, causes increased risk of fractures due to loss of bone desnity and falls [3], [4], [5], [6], [7], [8], [9]. The inter-relationship of stroke, physical inactivity, osteoporosis and fracture is mutually implicative and constitutes a vicious cycle for the elderly. An intervention that ameliorates any of these four components may significantly promote health in this population. Vertebroplasty is now a common surgical procedure for osteoporotic vertebral fractures. Aside from being popular among spine care specialists, its effect of pain relief has been demonstrated by randomized control studies [10], [11], [12]. Pulmonary, venous and cerebrovascular embolism with or without symptoms has been reported as complications [13], [14], [15], [16]. Moreover, the long-term effect of vertebroplasty regarding the incidence of stroke in osteoporotic patients remains elusive.

Although very rare, stroke can happen soon or long after percutaneous vertebroplasty in osteoporotic patients [13], [17], [18], [19]. There is increased risk of stroke after hip fractures and osteoporotic vertebral fractures [20]. This study hypothesized that the risk of stroke in osteoporotic patients did not increase after percutaneous vertebroplasty. The study also aimed to investigate the incidence and risk ratios of stroke after percutaneous vertebroplasty, as well as the differences in specific types (i.e. hemorrhagic or ischemic) of strokes.

In order to investigate such infrequent events and their correlation with surgery, a large number of patients with an extremely high follow-up rate was required. Thus, the National Health Insurance Research Database (NHIRD) was used so that follow-up would not be affected by patients seeking medical services across institutions. In Taiwan, hindrance to medical care is extremely low; this therefore enhanced the completeness and accuracy of identifying strokes, especially for those that did not happen immediately post-operation. This cohort study was deliberately designed to investigate the correlation of percutaneous vertebroplasty and subsequent strokes by applying a prudently matched comparison group with very similar disease profiles.

## Materials and Methods

### Data source

The National Health Insurance Research Database (NHIRD) of Taiwan contains 26 million administered insurants, accumulated between January 1996 and December 2008. A unique feature of the database is its comprehensive coverage of 99% of the population [21], who has unrestricted access to any healthcare provider of the patient's choice [22]. NHIRD data are de-identified and encrypted before their release for medical research. Thus, this study was exempted from full review by the Institutional Review Board.

The National Health Research Institute (NHRI) recompiles the medical claims and makes the database publicly available for research purposes. To protect privacy, individual and hospital identifiers are unique to the research database and can not be used to trace back to each individual patient or healthcare provider. Moreover, regular cross-checks and validation of the medical charts and claims are performed by the Bureau of National Health Insurance (NHI) of Taiwan to ensure the accuracy of diagnosis coding of the NHIRD. Fraudulent coding, overcharging, or malpractice by physicians and institutions are subject to penalties or suspension. Therefore, the fidelity of coding in the database is reliable; this was confirmed by a validation study [23].

### Study cohort

**Figure 1:**
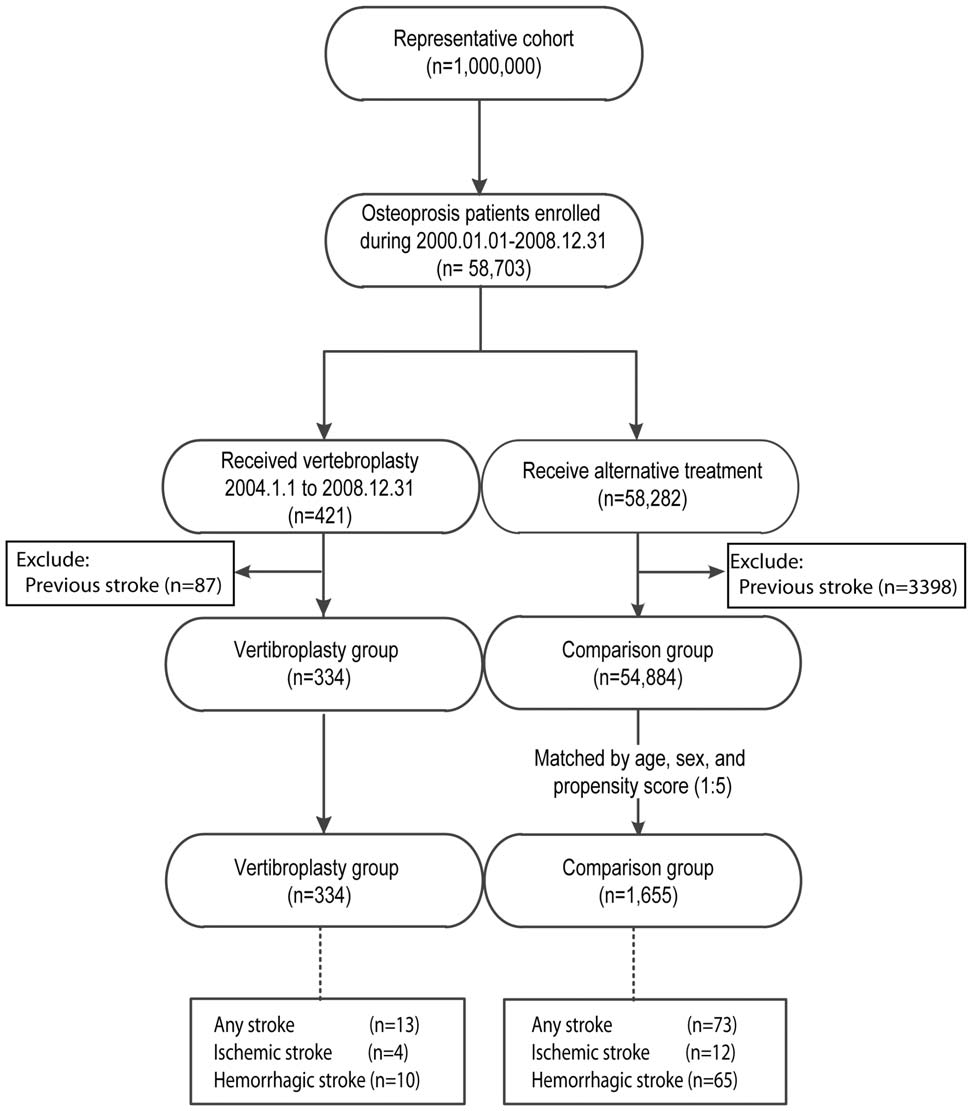
Flow of data processing. From an osteoporotic cohort of 58,703 patients in a nationwide representative cohort of one million people, 421 patients had percutaneous vertebroplasty (vertebroplasty group; n = 421), while age-, sex- and propensity score-matched patients comprised the comparison group (n = 1,655). The two groups were compared and followed-up for up to five years for subsequent stroke events.

This cohort study used a representative cohort composed of one million of Taiwan's cumulative population during the period January 1, 2000 to December 31, 2008. The representative cohort was provided by the NHRI through a random selection for scientific purposes, with no significant differences in age, gender or healthcare costs between the representative group and all beneficiaries under the NHI program. Many studies using this representative cohort have been published [20], [24], [25], [26]. Extraction of the vertebroplasty and comparison groups from the database is illustrated in Figure 1. All original claims data of the extracted subjects were analyzed.

### Identification of the vertebroplasty and medical treatment groups

A total of 58,703 persons with the International Classification of Disease, 9th Version (ICD-9) diagnostic code for osteoporosis (733.0) were identified from the database. First-time hospitalization with the ICD-9 procedure codes of percutaneous vertebroplasty (81.65) and percutaneous vertebral augmentation (81.66) from January 1, 2004 to December 31, 2008 were identified and enrolled as the vertebroplasty group. The first day of follow-up (entry date) was defined as the date of vertebroplasty.

A carefully designed one-to-five comparison was conducted. The osteoporotic cohort also included all enrollees not exposed to vertebroplasty during the study period. For every subject in the vertebroplasty group, five subjects who received medical treatment (but not vertebroplasty) were randomly assigned as the comparison control group. This group had prudently matched characteristics, (i.e., propensity scores), as described below.

Propensity scores (i.e., the predicted probability that a person would receive vertebroplasty) were used to capture the comparison group in order to adjust for confounding factors associated with receiving vertebroplasty. The propensity scores were calculated by medical co-morbidities (i.e., history of hypertension, diabetes, dyslipidemia, chronic obstructive pulmonary disease, chronic renal failure, and Parkinson's disease), demographic characteristics (e.g., age, sex, insurance level, geographic location of residence, and urbanization level of residence), and other spinal diseases (e.g. spondylosis, spondylolithesis, spinal stenosis, intervertebral disc disorders, and inflammatory spondylopathies).

The first matched visit date of those in the comparison group was designated as their first day of follow-up (entry date). Every subject in the study cohort was tracked back from enrolment date to January 1, 2000 to ensure no previous vertebroplasty or stroke events. Each subject was followed-up for up to five years. The cohort study censored follow-up only on the following conditions: when the subjects expired, on the dates of outcome incidence (i.e. stroke), or at the end of this cohort (December 31, 2008) (Fig. 1).

### Ascertainment of covariates

There are known and unknown risk factors of stroke [27], [28]. This study attempted to include influential covariates of stroke incidence for adjustment, including co-morbidities, exposure to medications, and baseline demographic characteristics. Co-morbidities included: hypertension (ICD-9 code, 401-5.x), diabetes mellitus (250.x), dyslipidemia (272.0-4), chronic obstructive pulmonary diseases (COPD) (490, 491.0-1, 491.20-2,491.8-9, 492.0, 492.8, 494, 494.0-1, and 496), chronic renal failure (585.x), Parkinson disease (332.0), atrial fibrillation (426-7.x), coronary heart disease (410-4.x), and valvular heart disease (394-7.x, 424.x). These were determined by the presence of either diagnostic codes of outpatient records or discharge codes of hospitalization records six months before the entry dates to the date of the outcome event or the end of follow-up. Adjustments for exposure to medications were defined as more than 90 days cumulative exposure to the medications of aspirin, lipid-lowering drugs, nitrates, anti-coagulants and non-steroid anti-inflammatory drugs (NSAIDs) between the entry dates and end of follow-up.

The demographic characteristics of each patient were adjusted by Charlson's co-morbidity index, age, sex and socio-economic status [29], [30], [31]. Socio-economic status was estimated by insurance level, and geographic location or residency, and urbanization level, used a similar method as previous NHIRD studies [22], [32], [33]. The income level of each subject was grouped into one of four categories according to the premium paid (NTD$ ≥40,000, 20,000–39,999, 1–19,999, and dependents). In the NHI of Taiwan, premiums are mostly determined by the insured wage and premium rate. Thus, the higher the premium, the implied higher income. Those without salaries, such as the unemployed, students, children or the elderly, are designated as dependents by the Bureau of NHI (BNHI), and the government or their foster families cover their insurance premiums.

The geographic locations of residency were grouped into four categories of northern, central, southern and eastern. More economic and political centers were located in the northern areas. The urbanization of the locations of NHI registration was also used as a proxy parameter for socio-economic status. According to previous reports using the NHIRD [34], urbanization levels are divided into 7 strata, in which level 1 is referred to as the “most urbanized” and level 7 as the “least urbanized”. However, in our study, given that there were fewer numbers in levels 5, 6 and 7, these three were combined into a single group and thereafter referred to as level 5 [31].

### Stroke incidences: hemorrhagic vs. ischemic

Any stroke event during the study period, determined by the date of hospitalization records with the discharge diagnostic code of stroke (ICD-9 code, 430–435) after the entry date, was identified. The first event of stroke was considered as the outcome in patients who had more than one stroke. To further analyze the characteristics of these strokes, all stroke events were further separated into hemorrhagic strokes, including sub-arachnoid hemorrhage (ICD- 9 code, 430) and intra-cerebral hemorrhage (ICD- 9 code, 431–432), and ischemic strokes (ICD- 9 code, 432–435).

### Statistical analysis

All of the data were linked using the SQL server 2008 (Microsoft Corp.) and analyzed by SPSS software (SPSS, Inc., Chicago, IL). Chi-square and independent t-tests were used to compare differences between the vertebroplasty and comparison groups. The Kaplan-Meier method and log-rank test were used to estimate the incidence rates of strokes. The Cox proportional hazard model with propensity scores was used to compare the stroke incidence rates between the two groups after adjusting for covariates. A two-tailed level of 0.05 was considered statistically significant.

## Results

A total of 1,989 patients with osteoporosis (i.e. 331 who received vertebroplasty and 1,655 who did not) were followed-up for 3,760.1 person-years. There were 74 ischemic and 16 hemorrhagic stroke events in 85 patients. The overall incidence rates of any, ischemic and hemorrhagic stroke were 22.6, 4.2 and 19.6 per 1,000 person-years, respectively.

### Similar demographics and co-morbidities

The gender distribution (78.1% female), mean age (75 years), and other parameters of socio-economic status were all very similar between the vertebroplasty and comparison groups (Table 1).

**Table 1:** Comparison for demographics and socio-economic status (n = 1989).

	Comparison group		Vertebroplasty group		
	n = 1655	(%)	n = 334	(%)	*p* value
Gender					0.975
Female	1292	(78.1)	261	(78.1)	
Male	363	(21.9)	73	(21.9)	
Age, Mean (SD)	75.1	(9.0)	75.0	(9.3)	0.859
Demographic characteristics					
Insurance levels (NTD$)					0.427
40,000-	20	(1.2)	2	(.6)	
20,000–39,999	625	(37.8)	139	(41.6)	
1–19,999	382	(23.1)	69	(20.7)	
Dependent	628	(37.9)	124	(37.1)	
Geographic locations					0.065
Northern area	712	(43.0)	165	(49.4)	
Central area	306	(18.5)	44	(13.2)	
Southern area	587	(35.5)	114	(34.1)	
Eastern area	50	(3.0)	11	(3.3)	
Urbanization levels					0.435
1 (most urbanization)	407	(24.6)	71	(21.3)	
2	409	(24.7)	75	(22.5)	
3	235	(14.2)	54	(16.2)	
4	294	(17.8)	68	(20.4)	
5 (least urbanization)	310	(18.7)	66	(19.8)	

The co-morbidities were similar between the two groups, including hypertension (*p* = 0.413), diabetes (*p* = 0.609), dyslipidemia (*p* = 0.964), chronic pulmonary obstructive disease (*p* = 0.288), chronic renal failure (*p* = 0.659), and Parkinson's disease (*p* = 0.163). There were no differences in the Charlson's co-morbidity index between the vertebroplasty and comparison groups (2.8 vs. 2.7, *p* = 0.358). Spinal diseases, including inflammatory spondylopathies, spondylosis, spondylolisthesis and intervertebral disc disorders, were also similar except for spinal stenosis, which was significantly more evident in the vertebroplasty group (21.0% vs. 15.2%, *p* = 0.009). There were no differences in the propensity scores between the vertebroplasty and comparison groups (0.56 vs. 0.58, *p* = 0.066) (Table 2).

**Table 2:** Comparison of co-morbidities (n = 1989).

	Comparison group		Vertebroplasty group		
	n = 1655	(%)	n = 334	(%)	*p* value
*Comorbidities*					
Hypertension					0.413
Yes	1157	(69.9)	241	(72.2)	
No	498	(30.1)	93	(27.8)	
Diabetes					0.609
Yes	679	(41.0)	132	(39.5)	
No	976	(59.0)	202	(60.5)	
Dyslipidemia					0.964
Yes	800	(48.3)	161	(48.2)	
No	855	(51.7)	173	(51.8)	
COPD					0.288
Yes	874	(52.8)	187	(56.0)	
No	781	(47.2)	147	(44.0)	
Chronic renal failure					0.659
Yes	141	(8.5)	26	(7.8)	
No	1514	(91.5)	308	(92.2)	
Parkinson's disease					0.163
Yes	62	(3.7)	18	(5.4)	
No	1593	(96.3)	316	(94.6)	
Charlson's comorbidity index, Mean (SD)	4.01	(2.7)	4.16	(2.8)	0.358
*Spinal diseases*					
Inflammatory spondylopathies					0.991
Yes	10	(.6)	2	(.6)	
No	1645	(99.4)	332	(99.4)	
Spondylosis					0.747
Yes	224	(13.5)	43	(12.9)	
No	1431	(86.5)	291	(87.1)	
Spondylolisthesis					0.194
Yes	182	(11.0)	45	(13.5)	
No	1473	(89.0)	289	(86.5)	
Spinal stenosis					0.009*
Yes	251	(15.2)	70	(21.0)	
No	1404	(84.8)	264	(79.0)	
Intervertebral disc disorders					0.462
Yes	199	(12.0)	45	(13.5)	
No	1456	(88.0)	289	(86.5)	
Propensity score, Mean (SD)	0.56	(0.2)	0.58	(0.2)	0.066

*p<0.05.

### Incidence of stroke

**Figure 2:**
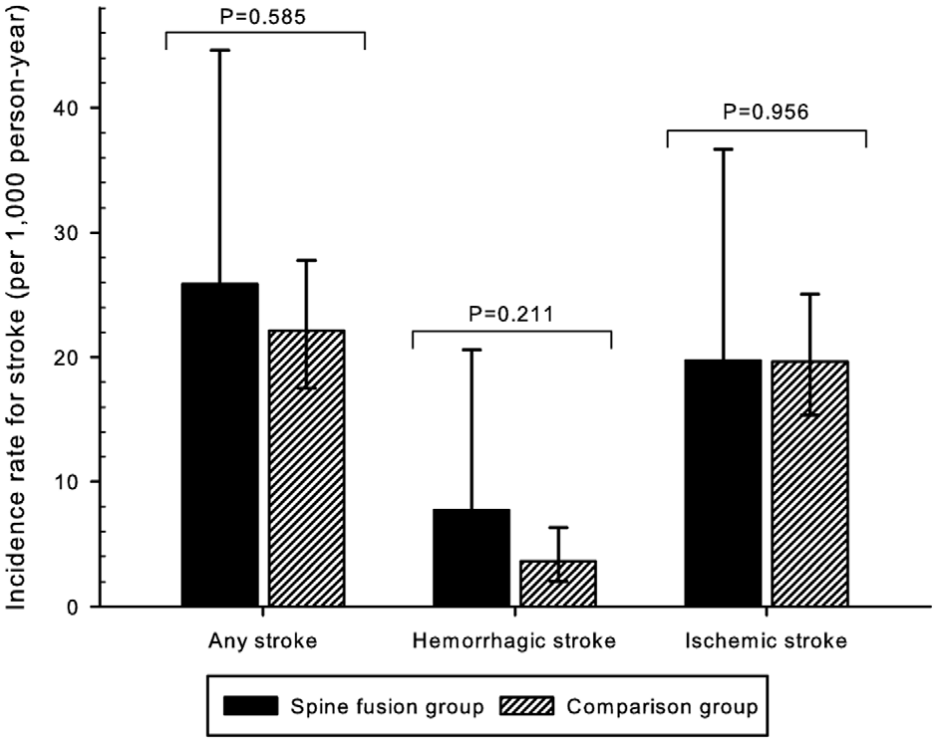
Comparison of stroke incidence rates. The vertebroplasty group had similar incidence rates of any, hemorrhagic and ischemic strokes to that of the comparison group.

There were no significant differences in stroke incidence rates after vertebroplasty among osteoporosis patients. Among the 334 subjects in the vertebroplasty group, the incidence rates (95% confidence interval) were 25.9 (15.0–44.6), 7.7 (2.9–20.6) and 19.7 (10.6–36.7) per 1,000 person-years for any stroke, hemorrhagic and ischemic strokes, respectively. On the other hand, for the 1,655 subjects without vertebroplasty in the comparison group, the incidence rates were 22.1 (17.5–27.8), 3.6 (2.0–6.3) and 19.6 (15.4–25.1) per 1,000 person-years for any stroke, hemorrhagic and ischemic strokes, respectively. There were no statistical differences in stroke incidence rates for stroke sub-types between the two groups (*p* = 0.585, 0.211, and 0.956, respectively) (Fig. 2).

**Figure 3:**
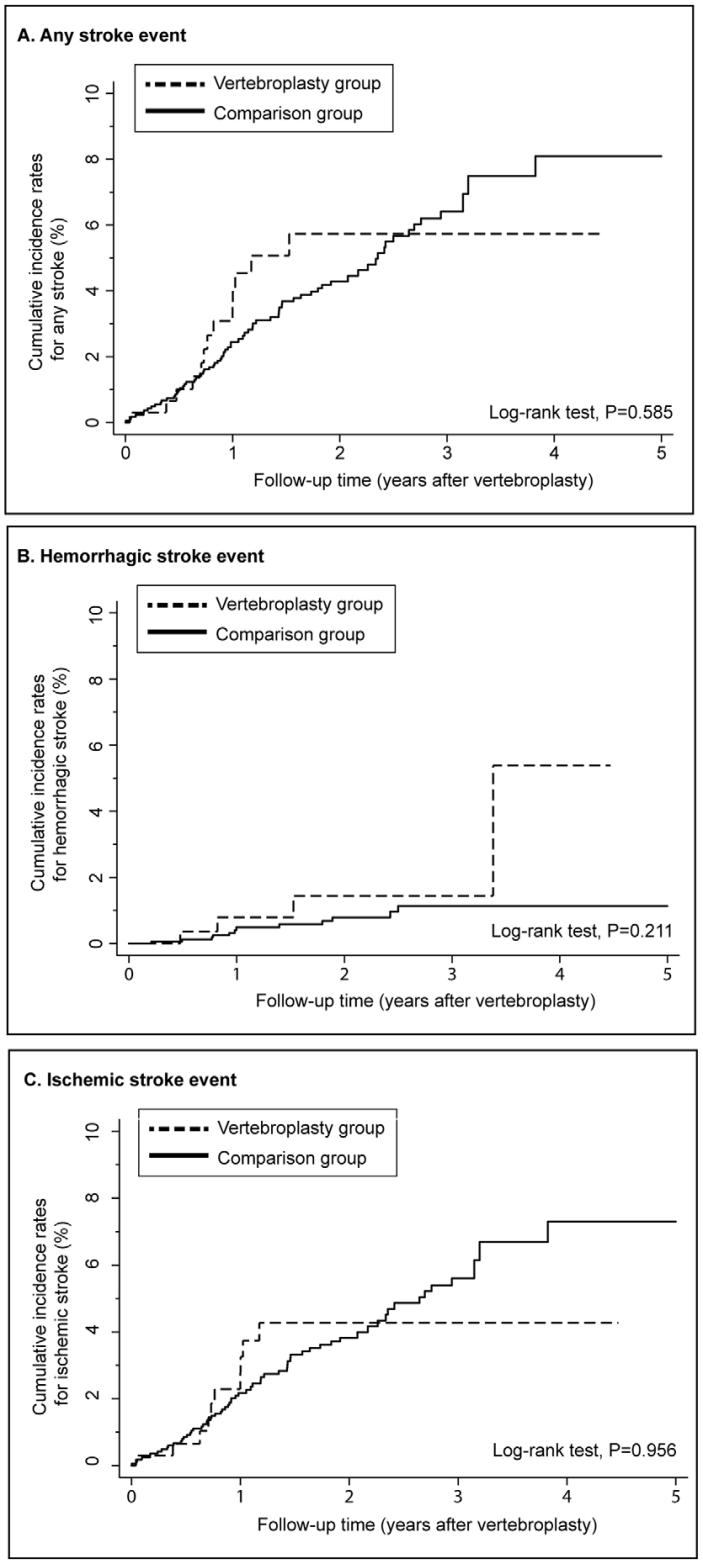
Cumulative incidence of stroke. The vertebroplasty group had similar cumulative incidences of (**A**) any stroke, (**B**) hemorrhagic stroke, and (**C**) ischemic stroke as the comparison group from the immediate post-operative period till the end of the five-year follow-up.

The accumulated incidence of each stroke sub-type showed no significant difference between the vertebroplasty and comparison groups (Fig. 3).

### Hazard ratios of strokes

Patients in the vertebroplasty group were insignificantly more or less likely to have stroke compared to the comparison group. The vertebroplasty group had crude hazard ratios (95% CI) of 1.13 (0.62–2.04), 2.21 (0.71–6.86), and 0.96 (0.49–1.91) for any stroke, hemorrhagic stroke and ischemic stroke (*p* = 0.693, 0.170, and 0.90, respectively) compared to the comparison group (Table 3). After adjustments for demographic characteristics, co-morbidities and medications, the adjusted hazard ratios of the vertebroplasty group were 1.22 (0.67–2.24), 3.17 (0.97–10.3), and 0.96 (0.49–1.91) for any stroke, hemorrhagic stroke and ischemic stroke (*p* = 0.518, 0.055, and 0.91, respectively). Thus, osteoporotic patients who received vertebroplasty did not have an increased risk of stroke in the five years post-operation.

**Table 3:** Hazard ratios of subsequent strokes post-vertebroplasty (2004.1.1–2008.12.31, n = 1989).

Stroke during 5-year-follow-up	Comparison group	Vertebroplasty group	(95% CI)	*p* value
Any stroke				
Crude hazard ratio	1.00	1.13	(0.62–2.04)	0.693
Adjusted hazard ratio^a^	1.00	1.22	(0.67–2.24)	0.518
Hemorrhagic stroke				
Crude hazard ratio	1.00	2.21	(0.71–6.86)	0.170
Adjusted hazard ratio^a^	1.00	3.17	(0.97–10.3)	0.055
Ischemic stroke				
Crude hazard ratio	1.00	0.96	(0.49–1.91)	0.90
Adjusted hazard ratio^a^	1.00	0.96	(0.49–1.91)	0.91

a Adjustments were made for demographic characteristics (i.e., age, sex, insurance level, geographic location, and urbanization level), co-morbidities (e.g., hypertension, diabetes, valvular heart disease, arrhythmia, cardiovascular disease and Charlson's co-morbidity index), medications (e.g., aspirin, nitrates, lipid lowering drugs, anti-coagulants, and NSAIDs), and baseline propensity scores.

## Discussion

In the last decade, percutaneous vertebroplasty has been accepted as an option for spinal compression fractures for elderly patients with osteoporosis. Numerous reports have demonstrated its rapid pain relief [10], [11], [35]. However, in spite of the escalating number of vertebroplasty procedures performed worldwide, there is a lack of robust evidence about the long-term effects [10], [11], [12], [36], [37], [38]. Stroke is rare after vertebroplasty but can cause severe neurologic consequences or death [17], [18], [19]. To date, the actual incidence and risk of stroke after percutaneous vertebroplasty remain elusive in the literature.

The current study used a comprehensive nationwide database, NHIRD, to investigate the risk and incidence of stroke in patients with osteoporosis, but who were treated differently. A total of 334 vertebroplasty patients and 1,655 medically treated patients were extracted from an osteoporotic cohort. In a follow-up of five years, the group of patients who had vertebroplasty was compared to the well-matched (i.e. age, sex and co-morbidities) group of patients not treated with vertebroplasty. There were no significant differences in the incidence and risk of stroke between the two groups. Thus, percutaneous vertebroplasty does not alter the chances of stroke in osteoporotic patients. This data also corroborates the safety of vertebroplasty and long-term medical outcome.

Several reports of prospective randomized control studies of vertebroplasty have been published recently [10], [11], [12]. Most clinicians agree that in selected patients with acute osteoporotic vertebral compression fractures and persistent pain, percutaneous vertebroplasty appears to be an effective and safe procedure. Pain relief is immediate and sustainable for at least a year, and is significantly greater than that achieved with conservative treatments [10]. However, the actual long-term outcome of vertebroplasty is uncertain. The most common concern of this procedure is the possible increased risk of vertebral compression fracture at adjacent spinal levels [39], [40]. Other effects of this procedure on the medical condition of osteoporotic patients who are particularly old and at a high risk of cardiovascular and other systemic diseases, are not addressed in the literature. This report is the first investigation focused on the risk of subsequent stroke after percutaneous vertebroplasty.

The complication rate of vertebroplasty is low but very serious, and irreversible complications have been reported, including spinal cord injury, nerve root compression, venous and pulmonary embolism, and cardiovascular collapse [13], [14], [15], [16], [35], [41], [42], [43]. The elderly, in whom most osteoporotic vertebral compression fractures occur, are particularly at higher risk of stroke. The database used in this study is uniquely appropriate to investigate the risks of stroke after vertebroplasty. Because the monopolistic government-operated health insurance system offers unlimited access to health care for the entire population of Taiwan and does not restrict health service providers, the universal coverage of health insurance yields an opportunity for an extremely high follow-up rate. This health insurance system even reimburses health care services provided by some overseas institutions. Theoretically, all subsequent stroke events were captured during the study period. Loss of follow up would only occur if the patient refused to seek medical attention at all upon having a stroke and still stayed in the system for a long time, which is very unlikely. Therefore, the incidence rates computed here are very accurate.

The strength of this report is its demonstration of the rarity and unlikelihood of stroke after vertebroplasty, even in patients with osteoporosis, who are older and potentially of a higher risk of stroke. The well-matched comparative group of patients ameliorates most of the confounders, rendering as valid this study that was specifically aimed at the correlation of vertebroplasty and stroke. The results indicating the safety of this procedure, although may be anticipated by most spine surgeons, is proven for the first time. One can infer that the increased risk of stroke brought on by osteoporotic spinal compression fracture is likely to be offset by this procedure. But this inference remains uncertain and its causes also need further investigation to corroborate.

Furthermore, after the strokes were divided into hemorrhagic stroke and ischemic stroke, there was still little difference between the patients who underwent vertebroplasty and others (Fig. 2). The Kaplan-Meier analysis and adjustment of all other confounding factors for stroke allowed for a valid estimation of the risks of any, hemorrhagic and ischemic strokes (Fig. 3). This information may be valuable in peri-operative management of vertebroplasty for elderly osteoporotic patients.

This study has several limitations. First, the detailed operative notes of vertebroplasty were not available for analysis. The levels of procedure, amount of cement injected, indications of surgery, and severity of pre-operative symptoms were not analyzed. Second, the comparison group was composed of osteoporotic patients with similar medical conditions but not treated by vertebroplasty; the percentage of acute vertebral compression fracture in the comparison group may not be as high as those treated by vertebroplasty. This issue could be a major difference between the groups (e.g. less severe osteoporosis could imply less likelihood of stoke). A randomized study of patients with acute osteoporotic compression fracture may overcome this limitation. Third, stroke is relatively rare (22.6 per 1,000 person-years) in these osteoporotic patients. Although the extremely high follow-up rate substantially compensated for this, a larger scale study with longer follow-up could further corroborate it.

In conclusion, osteoporotic patients who undergo percutaneous vertebroplasty are not at higher risk of any stroke in the five years after the surgery.
